# Beyond Scar Removal: Reprogramming the Glial Scar for Neural Repair

**DOI:** 10.3390/ijms27177553

**Published:** 2026-08-24

**Authors:** Chih-Wei Zeng

**Affiliations:** Department of Neuroscience, University of Texas Southwestern Medical Center, Dallas, TX 75390, USA; chih-wei.zeng@utsouthwestern.edu

**Keywords:** spinal cord injury, glial scar, neural regeneration, extracellular matrix, astrocytes, immunomodulation, biomaterials, stem cells, neuromodulation

## Abstract

Spinal cord injury (SCI) research has long treated the glial scar as the central obstacle to regeneration. That view is useful, but it is now too narrow. The scar is not a passive wall. It is an evolving repair tissue that contains inflammation, stabilizes the lesion, remodels extracellular matrix, and, under many conditions, restricts axonal growth. The next phase of SCI therapy should therefore move from scar suppression to scar reprogramming. In this Opinion, I argue that the field should prioritize temporally precise, spatially targeted, and biomarker-guided interventions that preserve the protective functions of the scar while reducing its chronic inhibitory features. Cell transplantation, biomaterials, neurotrophic factors, gene therapy, immunomodulation, and neuromodulation each offer important strengths, but none is sufficient as a stand-alone regenerative therapy. The most credible path forward is a staged combinatorial strategy that converts the lesion microenvironment into a permissive, instructive, and trainable tissue bridge.

## 1. Introduction: The Field Is Asking the Wrong Question

**Figure 1:**
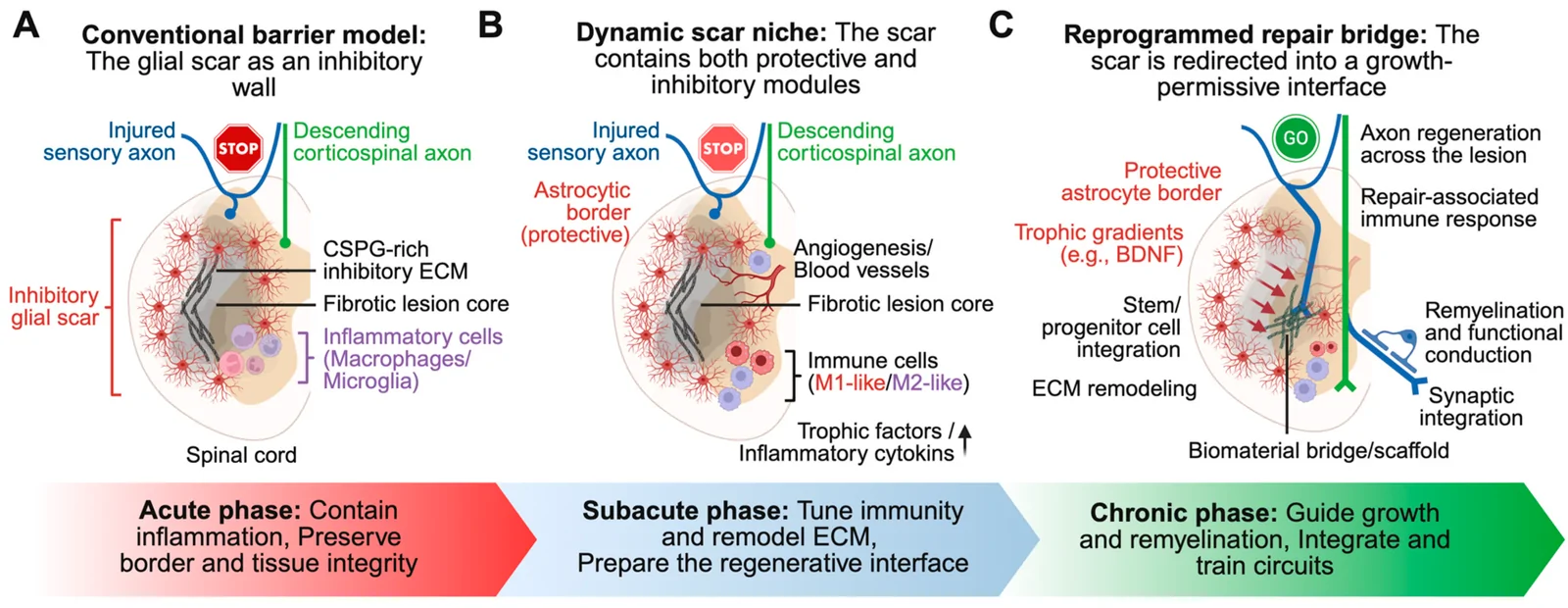
Reprogramming the glial scar after spinal cord injury: from inhibitory wall to repair bridge. (**A**) The conventional barrier model views the glial scar primarily as an inhibitory wall. Reactive astrocytes, CSPG-rich extracellular matrix, fibrotic lesion-core components, and inflammatory cells form a dense environment that restricts the extension of injured sensory and descending corticospinal axons. (**B**) The dynamic scar-niche model recognizes that the scar contains both protective and inhibitory components. The astrocytic border limits the spread of inflammation and stabilizes injured tissue, whereas fibrotic scarring, heterogeneous immune responses, altered vascular remodeling, trophic imbalance, and inflammatory signaling may create a non-permissive microenvironment. (**C**) Scar reprogramming seeks to preserve protective astrocytic functions while redirecting maladaptive components through ECM remodeling, localized trophic support, stem/progenitor cell integration, biomaterial bridging, immune modulation, remyelination, and synaptic integration. The lower timeline summarizes predominant therapeutic priorities across overlapping SCI phases: acute inflammatory containment and tissue stabilization; subacute immune and ECM remodeling with preparation of the regenerative interface; and chronic target-specific axonal growth, remyelination, circuit integration, neuromodulation, and rehabilitation. These phases are conceptual and should not be interpreted as rigid therapeutic windows. Created in BioRender. Zeng, C. (2026) https://BioRender.com/qkbh2qi.

The failure of meaningful regeneration after spinal cord injury (SCI) is often explained through a familiar image: axons encounter the glial scar, lose their ability to extend, and functional recovery stalls. This image is powerful because it captures a real biological problem. After SCI, reactive astrocytes, microglia, macrophages, pericyte-derived stromal cells, fibroblasts, and extracellular matrix (ECM) molecules reorganize around the lesion. Chondroitin sulfate proteoglycans (CSPGs), tenascins, hyaluronan, fibronectin, and other matrix components accumulate in ways that can suppress neurite outgrowth and limit circuit reconnection [1,2,3,4,5]. Yet, the image is also misleading. A scar is not simply debris that should be cleared away. It is a biological decision point. In the acute phase, glial and stromal responses restrict the spread of inflammatory and cytotoxic damage, restore a boundary between injured and preserved tissue, and provide mechanical stabilization. Removing or broadly suppressing these responses can make injury worse. The more important question is not how to eliminate the scar, but how to convert it from a chronic inhibitory boundary into a controlled regenerative interface [6,7,8]. This shift in framing should now define the field. This conceptual shift from scar removal to scar reprogramming is summarized in Figure 1.

## 2. Why the Glial Scar Should Be Treated as an Active Therapeutic Target

The strongest lesson from recent SCI biology is that the glial scar is heterogeneous across time, space, and cell type. Reactive astrocytes are not one population with one function. Microglia and macrophages do not exist as a simple M1 versus M2 binary. ECM remodeling is not uniformly hostile. TGF-β, JAK-STAT, NF-κB, RhoA/ROCK, Wnt/β-catenin, and other pathways produce context-dependent effects that vary with lesion severity, anatomical level, timing, age, and immune status [9,10,11,12,13]. Emerging single-cell and spatially resolved approaches now provide a means to resolve this heterogeneity at substantially greater depth. Single-cell and single-nucleus transcriptomic analyses can identify injury-associated states across astrocytes, microglia, macrophages, stromal cells, vascular cells, oligodendroglial populations, and neurons that may be obscured by bulk-tissue measurements, whereas spatial transcriptomics can localize these programs relative to the lesion core, astrocytic border, spared tissue, vascular compartments, and regenerating pathways [11,14]. These multidimensional maps may help define when, where, and in which cell populations scar-reprogramming interventions should be deployed and may support biomarker development and future patient stratification. However, transcriptional identity should not automatically be equated with functional phenotype; candidate cell states and signaling programs require validation through perturbation, anatomical analysis, electrophysiology, and behavioral testing.

This complexity is not a weakness of the field. It is a design principle. A successful SCI therapy must preserve early containment, limit secondary injury, reduce chronic inhibitory ECM, promote directed axonal extension, restore myelination, and train spared and newly formed circuits. No single intervention can accomplish all of these tasks. Chondroitinase ABC can reduce CSPG-mediated inhibition, but ECM digestion alone does not guarantee appropriate axon targeting [5,15,16]. Neurotrophic factors can promote growth, but uncontrolled gradients risk aberrant sprouting, pain, or maladaptive plasticity [17,18]. Stem cell grafts can supply relays, trophic support, and new cellular substrates, but they require survival, vascular support, immune compatibility, and precise integration. Electrical stimulation can re-engage dormant circuits, but improved output does not automatically prove structural regeneration [19,20,21,22]. These are not reasons for pessimism. They are reasons to abandon one-target thinking.

## 3. The Major Strength of Current SCI Research Is the Expanding Toolbox

The SCI field has never had more therapeutic options. Immunomodulatory strategies can temper secondary damage and shift inflammatory phenotypes toward repair. Biomaterials can provide structural support, local drug delivery, stiffness matching, and spatial organization. Stem and progenitor cells can replace lost cellular elements, supply trophic signals, and establish relay circuits. Gene therapy may be particularly valuable within this modular framework because it can provide sustained, transient, cell-selective, or anatomically restricted control over multiple components of the lesion microenvironment. For example, astrocyte-selective adeno-associated virus (AAV) delivery of ADAMTS4 has been used to remodel CSPG-rich ECM and, when paired with hindlimb rehabilitation, improve recovery after experimental SCI [23]. Non-viral mRNA delivery offers complementary temporal control: local BDNF mRNA delivery has provided transient neurotrophic support after contusive SCI [24], whereas methylprednisolone-substituted lipid nanoparticles carrying C3 transferase mRNA have combined local RhoA inhibition with modulation of the inflammatory environment [25]. Viral and RNA-based systems could therefore deliver neurotrophic, ECM-modifying, or immunoregulatory payloads within staged scar-reprogramming protocols. However, vector tropism, dose, duration of expression, immunogenicity, off-target effects, and reversibility remain critical translational constraints. Pharmacological strategies can target inhibitory signaling, including RhoA/ROCK and related pathways. Neuromodulation and rehabilitation can exploit neuroplasticity and reinforce functional circuits [26,27,28,29,30,31,32].

This toolbox is the field’s greatest strength. It makes it plausible to design therapies that are modular rather than monolithic. A rational regenerative protocol could begin with acute neuroprotection and immune containment, proceed to selective ECM remodeling, introduce a scaffold or cellular bridge when the lesion is biologically receptive, deliver neurotrophic cues in a controlled spatial pattern, and then use activity-dependent rehabilitation and stimulation to refine synaptic integration. In this model, the glial scar is not the enemy. It is the substrate to be engineered. Recent work reinforces the need to integrate materials science, cell therapy, and scar biology. Stiffness-optimized scaffolds can influence foreign body reaction, angiogenesis, and axonal growth [33]. GelMA-encapsulated iPSC-derived human spinal cord organoids provide an example of cell-based repair being paired with a defined biomaterial matrix [22]. Nano-primed reprogrammed neurons further illustrate how biomaterial delivery and cell-state conversion may reshape the scar microenvironment rather than merely bypass it [34].

## 4. The Major Weakness Is Temporal Imprecision

The field’s greatest weakness is that many interventions are still tested as if SCI were a static disease. It is not. SCI is a sequence of overlapping phases: hemorrhage, excitotoxicity, edema, immune infiltration, cell death, scar consolidation, demyelination, plasticity, and chronic remodeling. A treatment that is beneficial at one time point may be harmful at another. Broad anti-inflammatory therapy, for example, may reduce early tissue destruction but also suppress macrophage-mediated clearance and repair. Early ECM degradation may open growth channels but also destabilize the lesion. Late trophic delivery may promote sprouting, but without guidance it may fail to produce useful circuit formation [35,36]. Persistent epigenetic and metabolic changes in microglia may also contribute to excessive scar formation, which argues for timing-specific rather than globally suppressive immune intervention [13].

This timing problem is one reason why translation from animal models to patients remains disappointing. Preclinical studies often use controlled lesions, young animals, narrow time windows, and outcomes that do not fully reflect human disability. Human SCI is heterogeneous: cervical and thoracic injuries differ, contusion and compression differ, complete and incomplete injuries differ, and chronic lesions have different biology from acute lesions. Recent middle-aged mouse data further emphasize that injury biology and regenerative capacity must be interpreted in the context of age and host state [37]. The field needs fewer claims that a therapy promotes regeneration and more evidence defining when, where, in whom, and by what mechanism a therapy changes the lesion microenvironment [38,39].

## 5. Combination Therapy Is Necessary, but Not Every Combination Is Rational

It has become common to say that SCI will require combinatorial therapy. I agree, but the phrase is sometimes used too loosely. Combining two promising treatments is not a strategy. It is a hypothesis. A strong combination must specify which barrier each component addresses and why the order matters. The need for biological sequencing is consistent with a recently proposed sequential multimodal framework in which early localized immunomodulation precedes structural realignment and temporary ECM reconstruction, followed by sustained molecular guidance and matrix remodeling during axonal elongation [40]. This framework reinforces the principle that therapeutic order is itself a biological variable rather than a logistical preference. The scar-reprogramming model proposed here extends this logic by placing the evolving lesion niche at the center of treatment design: early protective functions should be preserved, whereas inflammatory, ECM, cellular, and structural components should be progressively redirected as the lesion becomes receptive to regeneration.

A rational scar-programming approach would include at least four modules. First, an immune module should limit secondary injury without abolishing repair-associated immune functions. Second, an ECM module should reduce chronic inhibition while preserving structural integrity and cell anchorage. Third, a growth, relay, and conduction module should promote endogenous axon regeneration, exogenous neural relay formation, or both, while also supporting target-specific guidance, appropriate synaptic integration, and remyelination [41,42,43]. Fourth, an activity module should use stimulation and rehabilitation to select, strengthen, and stabilize useful connections. Biomaterials are particularly attractive because they can integrate several modules: they can localize cytokines, enzymes, growth factors, extracellular vesicles, and cells within a defined lesion architecture. Table 1 summarizes this preservation-versus-redirection framework.

The most promising future therapies will not be defined by their ingredient list. They will be defined by biological logic. For example, a scaffold seeded with lineage-specified neural progenitors and loaded with temporally released ECM-modifying enzymes is more compelling if paired with neurotrophic gradients and task-specific rehabilitation. Similarly, neuromodulation becomes more powerful when used not only to produce movement, but to shape graft-host synapses and reinforce spared descending pathways. Mechanical control of fibrotic scar patterning also suggests that fibrosis may be redirected into a more growth-permissive architecture rather than simply removed [44]. The point is not to maximize complexity. The point is to match complexity to the biology of SCI [42,43,45,46].

## 6. Priorities Should Be Ranked, Not Merely Listed

A useful opinion must also be willing to rank priorities. In my view, three priorities should sit above the search for another single molecular target. First, the field should define the lesion state before applying therapy. An acute hemorrhagic lesion, a subacute inflammatory lesion, and a chronic cystic lesion are not the same biological problem. Second, the field should focus on anatomical specificity. Regeneration of the corticospinal tract, propriospinal relays, sensory pathways, and autonomic circuits will require different guidance rules. Third, the field should treat rehabilitation as a biological intervention, not as an optional clinical add-on. Activity changes gene expression, synaptic strength, myelination, and circuit selection. It should be built into preclinical design from the beginning rather than appended after a graft or drug has already been tested [21,47,48].

One cultural change is also needed. SCI research should reward negative and boundary-defining studies. Knowing that a given cell product fails in chronic cystic lesions, that a growth factor dose causes pain, or that a biomaterial works only within a narrow stiffness range is not failure. It is translational information [33]. The field has been slowed by optimism that is not always paired with reproducibility, dose–response analysis, and long-term safety assessment. Stronger reporting standards, blinded outcome assessment, sex-balanced studies, and replication across laboratories will make the next generation of therapies more credible [38]. There are also real risks that should be discussed more openly. Stem cell transplantation can generate ectopic growth, inappropriate differentiation, immune complications, or tumorigenic concerns, especially when pluripotent sources are used. Growth factor delivery can produce non-specific sprouting and pain if dose, gradient, and duration are poorly controlled. Immunomodulation can become immunosuppression. Matrix digestion can create permissiveness without direction. Neuromodulation can improve performance through compensation rather than true repair. These weaknesses should not weaken enthusiasm. They should make the field more rigorous [32,49]. A therapy that improves function through compensation may still be valuable, but it should not be mislabeled as regeneration.

For this reason, the best future studies will combine structural, molecular, electrophysiological, and behavioral evidence. Histology alone is insufficient. Behavioral recovery alone is ambiguous. Transcriptomics without function is descriptive. A convincing regenerative therapy should show that axons grow into a permissive substrate, form appropriate synapses, conduct signals, integrate with host circuitry, and improve functions that matter. The evidentiary bar should be high because the clinical need is high. An equally important challenge begins after an axon traverses the lesion. Scar penetration is an anatomical milestone, not a functional endpoint. Regenerating axons must reach appropriate target regions, establish suitable synaptic partners, and, where required, undergo sufficient remyelination to support reliable conduction and incorporation into circuits that contribute to meaningful behavior [41,42,43]. Growth without adequate guidance may instead produce ectopic or maladaptive connectivity, with potential consequences including neuropathic pain, spasticity, autonomic dysregulation, or pathological hyperexcitability [18]. Future studies should therefore determine not only how many axons cross the lesion, but also where they terminate, which cells they contact, whether their synapses are functionally active, whether conduction is restored, and whether activity-dependent training can refine the resulting circuitry. In this sense, the ultimate challenge is not simply scar versus axon, but reconstruction of functionally organized connectome architecture.

## 7. Patient-Centered Outcomes Must Discipline the Science

SCI research often overvalues motor scores and undervalues outcomes that patients identify as life changing. Hand function, trunk stability, autonomic regulation, bladder and bowel control, sexual function, pain, spasticity, fatigue, respiratory function, and independence may matter as much as walking. A therapy that improves segmental hand function after cervical SCI may be more meaningful than one that produces modest hindlimb stepping in a rodent model. A therapy that worsens neuropathic pain while improving axonal sprouting should not be celebrated as a regenerative success. This is where biomarkers and patient stratification become essential. Candidate biomarker strategies should combine complementary measures of lesion architecture, tissue injury, preserved connectivity, and functional state. Structural MRI can quantify lesion extent and residual midsagittal tissue bridges; in a multicenter cervical SCI cohort, tissue-bridge width improved prediction of short- and long-term sensorimotor recovery beyond clinical variables alone [50]. Serum and cerebrospinal fluid neurofilament light chain (NF-L) and glial fibrillary acidic protein (GFAP) are candidate indicators of axonal and astroglial injury, respectively, and higher acute concentrations have been associated with greater injury severity and worse neurological outcome [51]. Electrophysiological measures provide complementary evidence of preserved or restored conduction: motor-evoked potentials correlate with early neurological recovery after acute SCI, whereas lower-extremity somatosensory-evoked potentials have shown prognostic value in complete traumatic cervical SCI [52,53]. Inflammatory cytokine profiles, ECM-remodeling markers, immune phenotyping, and digital functional measures may provide additional information about lesion state and treatment response, but their utility for selecting scar-reprogramming interventions remains to be established. Future trials will likely require multimodal biomarker panels rather than a single marker to define lesion phase, preserved connectivity, inflammatory state, and response to therapy.

Trial design should also distinguish restorative, compensatory, and assistive effects. Epidural stimulation, brain–spine interfaces, robotic training, and pharmacological neuromodulation may substantially improve performance even when they do not rebuild long tracts. That distinction should be welcomed, not blurred. Patients need useful function, but scientists need mechanistic clarity. Future trials should therefore report whether gains arise from regenerated axons, strengthened spared pathways, improved excitability, compensatory strategies, or external assistance. Clear mechanistic categories will make combination therapies easier to optimize and will prevent premature claims that undermine confidence in the field [21,45,46,48,54]. The increasing complexity of regenerative interventions also creates important translational and ethical obligations. Multimodal protocols that combine cells, biomaterials, gene delivery, immunomodulation, and neuromodulation may introduce cumulative or interacting risks that are difficult to predict or attribute to a single component. Clinical translation should therefore include component-specific and combination-level safety assessment, manufacturing consistency, consideration of reversibility, and long-term surveillance, particularly for invasive or persistent interventions. Informed consent should clearly distinguish evidence of biological regeneration from the likelihood of meaningful functional benefit and should communicate uncertainty regarding durability, adverse effects, and the possibility of maladaptive circuitry. These principles are consistent with established guidance for the responsible clinical translation of stem-cell-based interventions [55]. The ambition to reconstruct neural circuitry must therefore be balanced with proportional risk, patient-defined priorities, and long-term safety.

## 8. Conclusions: The Glial Scar Is Not a Wall, but a Programmable Interface

The next leap in SCI therapy will not come from declaring victory over the glial scar. It will come from understanding the scar well enough to instruct it. The strongest available evidence supports a balanced view: the scar protects, inhibits, organizes, isolates, and signals. Its role changes over time. A field that tries only to remove it will miss its therapeutic potential.

I therefore argue for a deliberate shift from anti-scar therapy to scar-programming therapy. The goal should be to preserve early protective containment, reduce chronic biochemical inhibition, create permissive bridges, guide axons toward appropriate targets, and train circuits through activity. Importantly, successful lesion crossing should not itself define regenerative success. Newly generated or redirected axons must reach appropriate targets, become sufficiently remyelinated where necessary, form physiologically meaningful synapses, and be refined through activity without generating maladaptive circuitry [41,42,43]. Scar reprogramming should therefore be understood as one stage within the broader reconstruction of functional connectome architecture. This will require staged combinatorial interventions, robust biomarkers, clinically relevant models, and patient-centered endpoints. The glial scar should no longer be treated as a barrier at the edge of regeneration. It should be treated as the central engineering problem of spinal cord repair.

**Table 1:** **Opinion-guided framework for glial scar reprogramming after spinal cord injury.** The glial scar should be viewed as a dynamic injury niche. Therapeutic strategies should preserve protective scar functions while redirecting maladaptive inflammatory, fibrotic, and ECM-associated barriers.

Scar Module	Biological Tension	Reprogramming Direction	References
Astrocytic border	Contains inflammation and stabilizes the lesion, but may enrich inhibitory matrix.	Preserve the border; tune reactive astrocyte states.	[4,6,7,12]
Fibrotic lesion core	Supports wound closure, but excessive stromal scarring blocks growth.	Limit excessive fibrosis; organize the lesion core.	[3,12,13,56]
Immune compartment	Clears debris and coordinates repair, but chronic cytokines amplify damage.	Shift toward resolution-skewed immune responses.	[35,57]
Extracellular matrix	Provides structure, but CSPGs, tenascins, and hyaluronan restrict axons.	Remodel inhibitory ECM rather than erase the scar.	[3,19,58]
Trophic and guidance cues	Support neuronal survival and plasticity, but endogenous cues are weak or diffuse.	Deliver localized BDNF, GDNF, or NT-3 gradients.	[17,18,43]
Cell and scaffold interface	May bridge cavities, but graft survival and host integration remain limited.	Combine stem/progenitor cells with permissive biomaterials.	[20,27,33,47]
Integration and conduction	Axons may cross the lesion but terminate incorrectly, remain insufficiently myelinated, or form maladaptive circuits.	Guide target-specific growth; promote synaptic integration and remyelination; verify conduction and circuit safety.	[41,42,43]
Timing and activity	Repair depends on injury phase and circuit plasticity.	Sequence scar modulation with stimulation and rehabilitation.	[29,37,38,54]

## Data Availability

No new data were created or analyzed in this study. Data sharing is not applicable to this article.

## Abbreviations

AAV	Adeno-associated virus
BDNF	Brain-derived neurotrophic factor
CSPGs	Chondroitin sulfate proteoglycans
ECM	Extracellular matrix
GelMA	Gelatin methacryloyl
GFAP	Glial fibrillary acidic protein
GDNF	Glial cell line-derived neurotrophic factor
iPSC	Induced pluripotent stem cell
JAK-STAT	Janus kinase/signal transducer and activator of transcription
MEP	Motor-evoked potential
MRI	Magnetic resonance imaging
mRNA	Messenger RNA
NF-κB	Nuclear factor kappa-light-chain-enhancer of activated B cells
NF-L	Neurofilament light chain
NT-3	Neurotrophin-3
SCI	Spinal cord injury
TGF-β	Transforming growth factor-beta
Wnt/β-catenin	Wingless-related integration site/beta-catenin signaling
